# Validation of the FIB4 index in a Japanese nonalcoholic fatty liver disease population

**DOI:** 10.1186/1471-230X-12-2

**Published:** 2012-01-05

**Authors:** Yoshio Sumida, Masato Yoneda, Hideyuki Hyogo, Yoshito Itoh, Masafumi Ono, Hideki Fujii, Yuichiro Eguchi, Yasuaki Suzuki, Noriaki Aoki, Kazuyuki Kanemasa, Koji Fujita, Kazuaki Chayama, Toshiji Saibara, Norifumi Kawada, Kazuma Fujimoto, Yutaka Kohgo, Toshikazu Yoshikawa, Takeshi Okanoue

**Affiliations:** 1 Center for Digestive and Liver Diseases, Nara City Hospital, Nara, Japan; 2 Division of Gastroenterology, Yokohama City University Graduate School of Medicine, Yokohama, Japan; 3 Department of Medicine and Molecular Science, Graduate School of Biomedical Sciences, Hiroshima University, Hiroshima, Japan; 4 Department of Gastroenterology and Hepatology, Kyoto Prefectural University of Medicine, Kyoto, Japan; 5 Department of Gastroenterology and Hepatology, Kochi Medical School, Kochi, Japan; 6 Department of Hepatology, Graduate School of Medicine, Osaka City University, Osaka, Japan; 7 Department of Internal Medicine, Saga Medical School, Saga University, Saga, Japan; 8 Division of Gastroenterology and Hematology/Oncology, Department of Medicine, Asahikawa Medical College, Asahikawa, Japan; 9 School of Biomedical Informatics, University of Texas Health Science Center at Houston, Houston, TX, USA; 10 Hepatology Center, Saiseikai Suita Hospital, Suita, Japan

## Abstract

**Background:**

A reliable and inexpensive noninvasive marker of hepatic fibrosis is required in patients with nonalcoholic fatty liver disease (NAFLD). FIB4 index (based on age, aspartate aminotransferase [AST] and alanine aminotransferase [ALT] levels, and platelet counts) is expected to be useful for evaluating hepatic fibrosis. We validated the performance of FIB4 index in a Japanese cohort with NAFLD.

**Methods:**

The areas under the receiver operating characteristic curves (AUROC) for FIB4 and six other markers were compared, based on data from 576 biopsy-proven NAFLD patients. Advanced fibrosis was defined as stage 3-4 fibrosis. FIB4 index was assessed as: age (yr) × AST (IU/L)/(platelet count (10^9^/L) × √ALT (IU/L))

**Results:**

Advanced fibrosis was found in 64 (11%) patients. The AUROC for FIB4 index was superior to those for the other scoring systems for differentiating between advanced and mild fibrosis. Only 6 of 308 patients with a FIB4 index below the proposed low cut-off point (< 1.45) were under-staged, giving a high negative predictive value of 98%. Twenty-eight of 59 patients with a FIB4 index above the high cut-off point (> 3.25) were over-staged, giving a low positive predictive value of 53%. Using these cutoffs, 91% of the 395 patients with FIB-4 values outside 1.45-3.25 would be correctly classified. Implementation of the FIB4 index in the Japanese population would avoid 58% of liver biopsies.

**Conclusion:**

The FIB4 index was superior to other tested noninvasive markers of fibrosis in Japanese patients with NAFLD, with a high negative predictive value for excluding advanced fibrosis. The small number of cases of advanced fibrosis in this cohort meant that this study had limited power for validating the high cut-off point.

## Background

Type 2 diabetes mellitus is associated with nonalcoholic fatty liver disease (NAFLD) in clinical practice. NAFLD includes a wide spectrum of liver diseases ranging from simple steatosis, which is usually a benign and non-progressive condition, to nonalcoholic steatohepatitis (NASH), which can progress to liver cirrhosis (LC) and hepatocellular carcinoma (HCC) in the absence of significant alcohol consumption [1-4]. Liver biopsy remains a reliable tool for the diagnosis of NASH [1,5,6], and the most sensitive and specific method for providing prognostic information. However, it may not be practical to perform liver biopsies in every patient with NAFLD to ascertain the presence of NASH [6]. Moreover, biopsies are associated with significant limitations such as pain, risk of severe complications, sampling errors [7,8], cost, and patient unwillingness to undergo invasive testing. Since it is not easy to distinguish simple steatosis from NASH in diabetes clinics, simple scoring systems to derive progressive NASH are required. Numerous noninvasive panels of tests have been developed to stage liver disease, including a combination of clinical and routine laboratory parameters, as well as specialized tests involving direct markers of fibrosis and elastography [9-20]. Of these, the BAAT (body mass index [BMI], age, alanine aminotransferase [ALT], triglycerides) [14], European liver fibrosis (ELF) score [10], Fibrotest (BioPredictive, Paris, France) [9], Fibroscan (Echosens, Paris, France) [12], acoustic radiation force impulse elastography (Mochida Siemens Medical System Co. Ltd., Tokyo, Japan) [15], hyaluronic acid (HA) [16,17], type IV collagen 7S [18], BARD (BMI, aspartate aminotransferase [AST]/ALT ratio [AAR], diabetes mellitus [DM]) [19], N (Nippon) score [20] and the NAFLD fibrosis score (NFS) [21] have been tested in subjects with NAFLD.

The FIB4 index was developed as a noninvasive panel to stage liver disease in subjects with human immunodeficiency virus and hepatitis C virus (HCV) co-infection [22]. It relies on patient age, AST, ALT, and platelet count, which are routinely measured and are thus available for virtually all subjects with liver disease. This index has also been independently validated in subjects with HCV infection alone [23]. It has recently been demonstrated that its performance characteristics for the diagnosis of advanced fibrosis in NAFLD are better than those of other similar panels that do not require additional testing [24]. However, 74% of the subjects enrolled in the study were Caucasian, and validation of the FIB4 index in other ethnic groups is required before it can be applied globally. In this study, we therefore aimed to assess the accuracy of the FIB4 index for predicting advanced liver fibrosis in a cohort of Japanese patients with NAFLD.

## Methods

### Patients

A total of 576 patients with well-characterized and liver-biopsy-confirmed NAFLD between 2002 and 2008 were enrolled from the Japan Study Group of NAFLD (JSG-NAFLD), which includes nine hepatology centers in Japan: Center for Digestive and Liver Diseases, Nara City Hospital; Division of Gastroenterology, Yokohama City University Graduate School of Medicine; Department of Medicine and Molecular Science, Graduate School of Biomedical Sciences, Hiroshima University; Department of Gastroenterology and Hepatology, Kochi Medical School; Department of Internal Medicine, Saga Medical School, Saga University; Department of Hepatology, Graduate School of Medicine, Osaka City University; Department of Gastroenterology and Hepatology, Kyoto Prefectural University of Medicine; Division of Gastroenterology and Hematology/Oncology, Department of Medicine, Asahikawa Medical College; and Hepatology Center, Saiseikai Suita Hospital. All patients were also involved in the previous JSG-NAFLD study [25].

The diagnosis of NAFLD was based on the following criteria: (1) liver biopsy showing steatosis in at least 5% of hepatocytes [26]; and (2) appropriate exclusion of liver diseases of other etiologies, including viral hepatitis, autoimmune hepatitis, drug-induced liver disease, primary biliary cirrhosis, biliary obstruction, hemochromatosis, Wilson's disease, or α-1-antitrypsin- deficiency-associated liver disease. Patients who consumed > 20 g alcohol per day and patients with evidence of decompensated LC or HCC were excluded. Written informed consent was obtained from all patients at the time of liver biopsy, and the study was conducted in accordance with the Helsinki Declaration [27]. The study protocol was approved by the ethical committee of Nara City Hospital in Nara, Japan.

### Anthropometric and laboratory evaluation

Venous blood samples were taken in the morning after a 12-h overnight fast. Laboratory evaluations in all patients included a blood cell count and measurement of AST, ALT, γ-glutamyl transpeptidase (GGT), cholinesterase (ChE), total cholesterol, triglyceride, high-density lipoprotein (HDL) cholesterol, albumin, fasting plasma glucose (FPG), immunoreactive insulin (IRI), and ferritin. These parameters were measured using standard clinical chemistry techniques. BMI was also calculated; obesity was defined as BMI > 25, according to the criteria of the Japan Society for the Study of Obesity [28]. Patients were assigned a diagnosis of DM if they had documented use of oral hypoglycemic medication, a random glucose level > 200 mg/dL, or FPG > 126 mg/dL [29]. Hypertension was defined as a systolic blood pressure ≥ 130 mmHg or a diastolic blood pressure ≥ 85 mmHg or by the use of antihypertensive agents. Dyslipidemia was defined as serum concentrations of triglycerides ≥ 150 mg/dL or HDL cholesterol < 40 mg/dL and < 50 mg/dL for men and women, respectively, or by the use of specific medication [30]. Based on a review of the literature, the following scores were calculated for each patient: FIB4 [22], AAR, AST to platelet ratio index (APRI) [31], age-platelet index (AP index) [32], BARD score [19], N score [20], and NFS [13]. The values for the upper limit of normal were set according to the International Federation of Clinical Chemistry: AST 35 U/L for men, 30 U/L for women, and were comparable to the values used in other analyses. The specific formulae used to determine these scores are shown in Table 1.

**Table 1:** Formulae for determining noninvasive marker panels for detection of liver fibrosis.

Formula	Equation	
**FIB4 index**	$\left(\text{Age}\;\left[\text{years}\right]\times \text{AST}\;\text{[IU/L]}\right)∕\left(\text{platelet count}\;\text{[1}{\text{0}}^{9}\text{/L]}\times \sqrt{\text{ALT[IU/L]}}\right)$	

**AST to ALT ratio (AAR)**	AST/ALT	

**AST to platelet ratio index (APRI) ^a^**	([AST/ULN]/platelet count [10^9^/L]) × 100	

**Age-platelet index (AP index)**	Age (years)	platelet count (10^9^/L)
	< 30 = 0	< 225 = 0
	30-39 = 1	200-224 = 1
	40-49 = 2	175-199 = 2
	50-59 = 3	150-174 = 3
	60-69 = 4	125-149 = 4
	≥ 70 = 5	< 125 = 5
	Score is the sum of two (0-10)	

**NAFLD fibrosis score**	-1.675 + 0.037 × age (years) + 0.094 ×BMI (kg/m^2^) + 1.13 × IFG/diabetes (yes = 1, no = 0) + 0.99 × AST/ALT - 0.013 × platelet count (× 10^9^/L) - 0.66 × albumin (g/dL).	

**BARD score**	Scale 0-4	
	BMI ≥ 28 kg/m^2 ^= 1 point	
	AST/ALT ≥ 0.8 = 2 points	
	Diabetes = 1 point	

**N (Nippon) score**	Scale 0-4	
	female sex = 1 point	
	older age (> 60 years) = 1 point	
	type 2 diabetes = 1 point	
	hypertension = 1 point	

BMI, body mass index; IFG, impaired fasting glucose; INR, international normalized ratio; ULN, upper limit of normal.*^a^*ULN for AST: 30 in women, 35 in men.

### Histologic evaluation

All patients enrolled in this study underwent percutaneous liver biopsy under ultrasonic guidance. The liver specimens were embedded in paraffin and stained with hematoxylin and eosin, and Masson's trichrome. The minimum biopsy size was 20 mm and the number of portal areas was 10. The liver biopsy specimens were reviewed by two hepatopathologists (T.O. and Y.S.) who were blinded to the clinical data. Fatty liver was defined as the presence of steatosis in at least 5% hepatocytes, while steatohepatitis was diagnosed by steatosis, inflammation, and hepatocyte ballooning [2,3,26]. The individual parameters of NASH histology, including fibrosis, were scored independently using the NASH Clinical Research Network (CRN) scoring system developed by the NASH CRN [26]. Advanced fibrosis was classified as stage 3 or 4 disease (bridging fibrosis or cirrhosis).

### Statistical analysis

**Figure 1:**
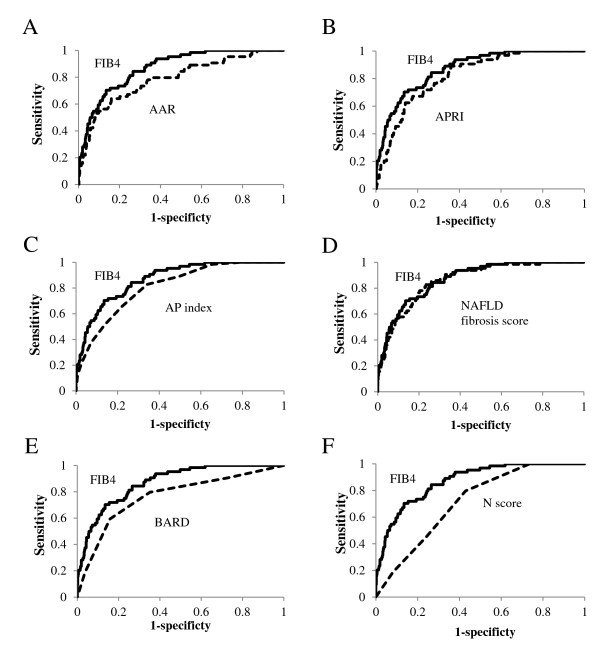
**Comparison of ROCs of FIB4 and other panels for the diagnosis of advanced fibrosis**.

Statistical analysis was conducted using SPSS 19.0 software (SPSS, Inc., Chicago, IL). Continuous variables were expressed as mean ± standard deviation (SD), or median (interquartile range). Qualitative data were presented as numbers with percentages in parentheses. Statistical differences in quantitative data were determined using the *t *test or Mann-Whitney U test. Fisher's exact probability test or χ^2 ^analysis was used for qualitative data (Table 2). The sensitivity and specificity for each value of each test were calculated to assess the accuracy of the clinical scoring system in differentiating between advanced and mild fibrosis, and receiver operating characteristic (ROC) curves were constructed by plotting the sensitivity against (1 - specificity) at each value (Figure 1). The diagnostic performances of the scoring systems were assessed by analysis of ROC curves. The most commonly used index of accuracy was the area under the ROC curve (AUROC), with values close to 1.0 indicating high diagnostic accuracy. (Table 3). The sensitivity, specificity, positive predictive value (PPV), and negative predictive value (NPV) were calculated for the two cut-off values (< 1.45 and > 3.25) proposed by Sterling [22] and those (< 1.30 and > 2.67) proposed by Shah [24]. Differences were considered statistically significant at *p *< 0.05.

**Table 2:** Characteristics of study population and values of noninvasive fibrosis marker panels^a^.

	Total (n = 576)	Fibrosis stage 0-2 (n = 512)	Fibrosis stage 3-4 (n = 64)	*p-value^b^*
**Age (yr)**	52.3 ± 15.4	51.2 ± 15.5	62.0 ± 10.1	< 0.0001
**Gender (female)**	280 (49%)	235 (46%)	45 (70%)	0.0003
**BMI (kg/m^2^)**	27.9 ± 4.9	27.8 ± 4.9	28.6 ± 4.8	0.2138
**Obesity (BMI > 25)**	418 (73%)	369 (72%)	49 (77%)	0.5524
**Hypertension (yes)**	184 (32%)	150 (29%)	34 (53%)	0.0062
**Type 2 diabetes (yes)**	241 (42%)	199 (39%)	42 (66%)	0.0001
**Hemoglobin (g/dL)**	14.6 ± 2.0	14.7 ± 2.0	13.7 ± 2.0	0.0001
**Platelet count (×10^9^/L)**	227 ± 67	235 ± 64	162 ± 52	< 0.0001
**AST (IU/L)**	43 (30-67)	41 (29-64)	61 (47-77)	< 0.0001
**ALT (IU/L)**	69 (43-112)	69 (43-69)	62 (46-94)	0.5074
**AST/ALT ratio**	0.65 (0.52-0.82)	0.63 (0.51-0.78)	0.98 (0.73-1.21)	< 0.0001
**GGT (IU/L)**	60 (39-99) (n = 572)	57 (36-92) (n = 508)	84 (59-128)	< 0.0001
**Albumin (g/dL)**	4.4 ± 0.4	4.4 ± 0.4	4.1 ± 0.4	< 0.0001
**Cholinesterase (IU/L)**	380 (330-433) (n = 527)	385 (337-439) (n = 466)	297 (244-367) (n = 61)	< 0.0001
**Total cholesterol (mg/dL)**	209 ± 40 (n = 467)	210 ± 39 (n = 409)	198 ± 42 (n = 58)	0.0484
**Triglyceride (mg/dL)**	147 (107-207) (n = 566)	150 (109-212) (n = 502)	131 (95-184) (n = 64)	0.0364
**HDL-C (mg/dL)**	50 ± 17 (n = 548)	50 ± 17 (n = 487)	51 ± 13 (n = 61)	0.7516
**LDL-C (mg/dL)**	128 ± 33 (n = 405)	129 ± 32 (n = 363)	120 ± 42 (n = 42)	0.1666
**Ferritin (ng/mL)**	173 (92-300)	169 (91-292)	216 (128-349)	0.0627
**FPG (mg/dL)**	103 (94-122) (n = 524)	103 (94-119) (n = 462)	111 (95-138) (n = 62)	0.0166
**IRI (μU/mL)**	11.6 (7.8-18.4)	11.3 (7.5-17.4)	17.3 (11.3-26.2)	< 0.0001
**FIB4 index**	1.23 (0.77-2.02)	1.13 (0.71-1.79)	3.17 (1.88-4.25)	< 0.0001
**AST/ALT ratio** **(AAR)**	0.65 (0.52-0.82)	0.63 (0.51-0.78)	0.98 (0.73-1.21)	< 0.0001
**AST to platelet ratio index (APRI)**	0.61 (0.40-0.98)	0.57 (0.38-0.92)	1.22 (0.86-1.79)	< 0.0001
**Age-platelet index (AP index)**	4 (2-6)	3 (2-5)	7 (5-8)	< 0.0001
**NAFLD fibrosis score**	-1.82 (-3.04 to -0.58)	-2.07 (-3.25 to -0.95)	0.25 (-0.60-1.06)	< 0.0001
**BARD score**				< 0.0001
**0**	144 (25%)	138 (27%)	6 (9%)	
**1**	201 (35%)	194 (38%)	7 (11%)	
**2**	112 (19%)	99 (19%)	13 (20%)	
**3**	88 (15%)	62 (12%)	26 (41%)	
**4**	31 (5%)	19 (4%)	12 (19%)	
**N score**				< 0.0001
**0**	135 (23%)	135 (26%)	0 (0%)	
**1**	170 (30%)	157 (31%)	13 (20%)	
**2**	118 (20%)	96 (19%)	22 (34%)	
**3**	99 (17%)	82 (16%)	17 (27%)	
**4**	54 (9%)	42 (8%)	12 (19%)	

BMI, body mass index; AST, aspartate aminotransferase; ALT, alanine aminotransferase; GGT, γ-glutamyl transpeptidase; HDL, high-density lipoprotein; LDL, low-density lipoprotein; FPG, fasting plasma glucose; and IRI, immuno-reactive insulin. *a *Values are mean  ±  SD, median (interquartile range), counts (%), as appropriate. *b*Values from univariate ordinal logistic regression, Mann-Whitney, or χ^2 ^analysis, as appropriate.

**Table 3:** Accuracy of noninvasive fibrosis marker panels.

Fibrosis panel	AUROC	Cut-off values	Sensitivity (%)	Specificity (%)	PPV (%)	NPV (%)
**FIB4 index**	0.871	1.45 3.25	90 48	64 95	24 53	98 94
**AST/ALT ratio (AAR)**	0.788	0.8 1	66 48	76 92	26 44	95 94
**AST to platelet ratio index (APRI)**	0.823	1	67	81	31	95
**Age-platelet index (AP index)**	0.810	6	66	78	27	95
**NAFLD fibrosis score**	0.863	-1.455 0.676	92 33	63 96	24 50	98 92
**BARD score**	0.765	2	80	65	22	97
**N score**	0.715	2	80	58	19	96

AUROC, area under the receiver operating characteristics curve; PPV, positive predictive value; NPV, negative predictive value

## Results

A total of 576 subjects were included in this analysis. Of these, 280 (49%) were women and 418 (73%) were obese (Table 2); 241 (42%) had type 2 DM and 184 (32%) were hypertensive. A total of 319 subjects had steatohepatitis, of whom 64 subjects had advanced fibrosis. As expected, subjects with more advanced fibrosis were significantly older, predominantly female, and more likely to be hypertensive, to have type 2 DM, to have higher AST, AAR, GGT, FPG, and IRI, and to have lower hemoglobin, platelet count, albumin, ChE, total cholesterol, and triglyceride. Regarding the individual components of the FIB4 score, the mean (± SD) or median [interquartile range] values were as follows: age (52.3 ± 15.4 years); AST (43 [30-67] IU/L); ALT (69 [43-112] IU/L), and platelets (227 ± 67 × 10^9^/L) (Table 2). The distribution of fibrosis stages included stage 0 (*n *= 263), stage 1 (*n *= 169), stage 2 (*n *= 80), stage 3 (*n *= 45), and stage 4 (*n *= 19). FIB4 values for the whole sample ranged from 0.17-10.74. The median FIB4 score was 1.23 (interquartile range, 0.77-2.02) (Table 3). The mean (interquartile range) FIB4 indices for stages 0, 1, 2, 3, and 4 were 1.09 (0.61-1.34), 1.40 (0.77-1.88), 2.36 (1.44-3.15), 3.23 (1.82-4.04), and 4.48 (3.19-5.17), respectively (*p *< 0.0001 by analysis of variance). The mean (interquartile range) FIB4 index was 1.13 (0.71-1.79) in patients with stage 0-2 fibrosis and 3.17 (1.88-4.25) in patients with stage 3-4 fibrosis (*p <*0.0001) (Table 2).

The sensitivity and specificity of FIB4 along the ROC were assessed first. At a sensitivity of 90% (FIB4 = 1.45) the specificity was 35%, while at a specificity of 90% (FIB4 = 2.67), the sensitivity was 52%. ROC curves were then developed for each of the noninvasive marker panels and superimposed, to determine which score would have the most clinical utility (Figure 1). ROC curves were created to determine the utility of the indices for predicting advanced fibrosis (stage 3 and 4 versus lower scores). The AUROC was greatest for FIB4 (0.871), followed by NFS (0.863), APRI (0.823), AP index (0.810), AAR (0.788), BARD score (0.765), and N score (0.715) (Table 3). As the NPVs for FIB4 index, AAR, APRI, AP index, NFS, BARD score, and N score were all greater than 95% using their lower cut-offs, these tests may have sufficient accuracy to be used clinically to exclude advanced fibrosis. Using this approach, a significant proportion of patients could avoid liver biopsy using each of these tests (Table 3). As the PPV were modest for all noninvasive tests, ranging from 19% to 53%, it was felt they were not accurate enough to be used as an alternative to liver biopsy. The PPV for FIB4 is highest among other noninvasive tests.

Using the low cut-off point proposed by Sterling and colleagues (< 1.45)[22], 330 of 336 (98.3%) patients without stage 3 or 4 fibrosis were correctly staged, while only 6 (1.7%) were under-staged (Table 4). All of the 6 patients with advanced fibrosis but FIB4 index below the low cut-off point had stage 3 fibrosis, none had stage 4 fibrosis. The NPV of this cut-off for stage 3 or 4 fibrosis was 98%. Using the high cut-off point proposed by Sterling and colleagues (> 3.25) [24], 31 of 59 (52.5%) patients with stage 3 or 4 fibrosis were correctly staged, while 28 (47.5%) were over-staged. Among the 28 patients without advanced fibrosis but FIB4 index above the high cut-off point, 18 had stage 2 fibrosis, 6 had stage 1, and 4 had no fibrosis. The PPV of this cut-off for stage 3 or 4 fibrosis was 53%. A total of 395 patients (69% of the cohort) had a FIB4 index < 1.45 or > 3.25; FIB4 identified the absence or presence of advanced fibrosis with 91% accuracy in these 361 subjects. A total of 181 subjects (31%) had FIB4 values in the indeterminate range (1.4-3.25).

**Table 4:** Proportion of patients who may potentially avoid liver biopsy using the simple non-invasive tests to exclude advanced fibrosis.

Fibrosis panel	Cut-off values	Patients avoiding liver biopsy*^a^*	False negative result
**FIB4 index**	< 1.45	336/576 (58%)	6 (2%)
	< 1.30	308/576 (53%)	4 (1%)
**AST/ALT ratio (AAR)**	< 0.8	413/576 (72%)	22 (5%)
**AST to platelet ratio index (APRI)**	< 1	435/576 (76%)	21 (5%)
**Age-platelet index (AP index)**	< 6	421/576 (73%)	22 (5%)
**NAFLD fibrosis score**	< -1.455	328/576 (57%)	5 (2%)
**BARD score**	< 2	355/576 (62%)	13 (4%)
**N score**	< 2	305/576 (53%)	13 (4%)

*^a^*Patients with a value below the cut-off.

On the other hand, using the low cut-off point proposed by Shah and colleagues (< 1.30) [24], 304 of 308 (99%) patients without stage 3 or 4 fibrosis were correctly staged, while only 4 (1%) were under-staged (Table 4). All of the 4 patients with advanced fibrosis but FIB4 index below the low cut-off point had stage 3 fibrosis and none had stage 4 fibrosis. The NPV of this cut-off for stage 3 or 4 fibrosis was 99%. Using the high cut-off point proposed by Shah and colleagues (> 2.67), 38 of 89 (43%) patients with stage 3 or 4 fibrosis were correctly staged, while 51 (57%) were over-staged. Among the 51 patients without advanced fibrosis but NAFLD fibrosis scores above the high cut-off point, 28 had stage 2 fibrosis, 14 had stage 1, and 9 had no fibrosis. The PPV of this cut-off for stage 3 or 4 fibrosis was 43%. A total of 397 patients (69% of the cohort) had a FIB4 index < 1.30 or > 2.67; FIB4 identified the absence or presence of advanced fibrosis with 86% accuracy in these 342 subjects. A total of 179 subjects (31%) had FIB4 values in the indeterminate range (1.30-2.67). Thus the prevalence of patients in the indeterminate range was similar using the two different cut-off values, but the number of patients with true positive or true negative predictions (accuracy) was higher using Sterling *et al*.'s cut-off values compared with Shah *et al*.'s (361 patients versus 342 patients). If liver biopsies were only performed in patients with an FIB4 index above the low cut-off point (> 1.45) proposed by Sterling, 336 (58%) of 576 biopsies could be avoided (Table 4).

The diagnostic accuracy of FIB4 index for detecting advanced fibrosis (stage 3-4) was also compared to that of NFS (Table 5). Three hundred and seventy patients (64% of the cohort) had an NFS <-1.455 or > 0.676; NFS identified the absence or presence of advanced fibrosis with 93% accuracy in these 344 subjects. A total of 206 subjects (36%) had NFS values in the indeterminate range (-1.455-0.676). Although the accuracy of NFS was higher (93%) than that of FIB4 (86%), more patients were correctly staged with FIB4 (*n *= 361) than with NFS (*n *= 344). Moreover, the percentage of patients in the undetermined range was lower for the FIB4 index (31%) than for NFS (36%). Using the cut-off values reported by Sterling and colleagues, discrepancies between FIB4 index and NFS were observed in 146 (39%) patients (Table 5). Patients were categorized into three groups, "low-risk" (< 10%), "intermediate-risk" (10-30%) and "high-risk" (> 30%), based on the combination of FIB4 index and NFS (Table 5). Only 1 patient (0.4%) of 243 patients with the low cut-off points for both FIB4 index and NFS had advanced fibrosis.

**Table 5:** Categorized risk groups for advanced fibrosis according to combined FIB4 index and NAFLD fibrosis score (NFS).

		FIB4 index (cut-off values proposed by Sterling et al.)			Total
		
		Low cut-off point (< 1.45)	Indeterminate (1.45-3.25)	High cut-off point (> 3.25)	
**NFS**	Low cut-off point (<-1.455)	283 [1 (0.4%)] ^a^	42 [4 (9.5%)] ^a^	3 [0 (0.0%)] ^a^	328 (56.9%) [5 (1.5%)]
	Indeterminate (-1.455-0.676)	53 [5 (9.4%)] ^a^	122 [19 (15.6%)] ^b^	31 [14 (45.2%)] ^c^	206 (35.8%) [38 (18.4%)]
	High cut-off point (> 0.676)	0	17 [4 (23.5%)] ^b^	25 [17 (68.0%)] ^c^	42 (7.3%) [21 (50.0%)]

**Total**		336 (58.3%) [6 (1.7%)]	181 (31.4%) [27 (14.9%)]	59 (10.2%) [31 (52.5%)}	576 (100%) [64 (11.1%)]

Total number of patients [stage 3-4 (%)]

Patients were categorized into three groups, "low-risk" (< 10%) ^a^, "intermediate-risk" (10-30%) ^b ^and "high-risk" (> 30%) ^c^, based on the combination of FIB4 index and NFS.

## Discussion

The AUROC of FIB4 was 0.871 for the diagnosis of advanced fibrosis, which was superior to those of the other noninvasive panels tested. For a value < 1.45, fibrosis could be excluded with 98% certainty (NPV 98%) whereas for a value > 3.25, the presence of significant fibrosis could be predicted with 53%. Despite the limited sensitivity of the FIB4 index in a population with a low prevalence of advanced fibrosis, the score was useful for ruling out advanced fibrosis. In our cohort, 58% of the liver biopsies could have been avoided if the procedure was not performed in patients with a FIB4 index below the low cut-off point (< 1.45). The score would therefore be particularly useful for reducing the number of unnecessary liver biopsies performed, and thus the costs of managing NAFLD patients in Asia, where advanced fibrosis is uncommon. A high cut-off FIB4 index of 2.67 which has been proposed by Shah and colleagues [24] had a low PPV (43%) in predicting stage 3 or 4 fibrosis. Our results contrast with those reported by Shah and colleagues [24], where a high cut-off FIB4 index of 2.67 had an 80% PPV in predicting stage 3 or 4 fibrosis; however the prevalence of advanced fibrosis in our study was only 11%, compared to 23% in Shah *et al*.'s study. Our study was therefore unable to reliably validate the high cut-off point, and larger Asian studies are warranted to investigate this. The FIB4 index was higher in our population than in Shah *et al*.'s study; stage 0-2: 1.13 (0.71-1.79) versus 0.97 (0.68-1.37), stage 3-4: 3.17 (1.88-4.25) versus 1.98 (1.28-3.08), probably because of older age, higher levels of ALT, and lower levels of platelets in our population.

The BARD score developed by Harrison *et al*. represents the weighted sum of three easily available variables (BMI ≥ 28 kg/m^2 ^[1 point], AAR ≥ 0.8 [2 points], and DM [1 point]), and the authors demonstrated that a score of 2-4 was associated with an odds ratio of 17 for predicting advanced fibrosis [19]. Although BARD score is simple to calculate, our validation study failed to detect any advantage of this score over FIB4; a BARD score of ≥ 2 was associated with a sensitivity, specificity, PPV and NPV for detecting advanced fibrosis of 80, 65, 22 and 97%, respectively. Consistent with the present study, Fujii and colleagues reported significantly poorer applicability of BARD in Japanese patients with NAFLD, compared with Caucasian subjects [33]. It has been suggested that BARD score is less predictive of advanced fibrosis in Japanese NAFLD patients because they are less obese than those in western countries. The N score (the total number of the following risk factors: female sex, age > 60 years, type 2 DM, and hypertension), which was established on the basis of data from 182 Japanese NAFLD patients in multiple centers in Nagasaki [20], requires no detailed laboratory measurements, but was not found to be superior to FIB4 index in our validation study. Angulo *et al*. found that the NFS, which consists of six variables (age, BMI, AAR, IFG/DM, platelet count, and albumin), reliably predicted advanced fibrosis in NAFLD patients [21]. In 428 (74%) of the subjects in the present study, FIB4 index was in accordance with NFS. The combination of two scoring systems could help to identify patients likely to have advanced fibrosis. Patients with FIB4 values above the high cut-off point (> 3.25) and NFS values above the low cut-off point (> -1.455) were at high risk (> 30%) for advanced fibrosis. If both FIB4 and NFS were applied to Japanese patients with NAFLD, patients with either FIB4 or NFS values below the low cut-off points (376/576, 65.3%) could avoid liver biopsies. In this way, when FIB4 was combined with NFS, its ability to predict or exclude advanced fibrosis improved further. In summary, the current study demonstrated that the FIB4 index, which can be established using a simple, relatively inexpensive method, correlated with the stage of fibrosis in adult subjects with NAFLD.

Type IV collagen is one of extracellular matrices that are produced by hepatic fibroblasts. The 7S domain in the N-terminus of type IV collagen is inserted in tissues and released into the blood by turnover in connective tissues. Therefore, the serum 7S domain level increases in parallel with the amount of fibrosis and in synthesis from stellate cells and myofibroblasts following increased liver fibrosis. In Japan, type IV collagen 7S is now widely used for assessing the extent of hepatic fibrosis in chronic liver diseases. Our data demonstrated that a cutoff point of 5.4 ng/ml provided a sensitivity and specificity of 86% and 87%, respectively, to detect advanced stage of NASH. The AUROC of type IV collagen 7s was: 0.926 for the diagnosis of advanced fibrosis, which was superior to FIB4 (data not shown). This data suggest that type IV collagen 7S is one of the best parameters among non-invasive parameters, but it costs too much to be determined routinely.

On the other hand, hepatic steatosis is frequently found in patients with HCV infection. Therefore, we also evaluated the value of FIB4 index in 185 HCV-infected patients with hepatic steatosis, including those with 72 advanced and 113 mild fibrosis. The AUROC of FIB4 was 0.808 for the diagnosis of advanced fibrosis. For a value < 1.45, fibrosis could be excluded with 89% certainty (NPV 89%) whereas for a value > 3.25, the presence of advanced fibrosis could be predicted with 82% (data not shown).

This study had several limitations. First, the proportion of subjects with advanced fibrosis was small, as reported in other Asian studies [34], and further Asian studies with more patients with advanced fibrosis are warranted. Second, patients were recruited from hepatology centers in Japan with a particular interest in studying NAFLD, and the possibility of some referral bias could therefore not be ruled out. Patient selection bias could also have existed, because liver biopsy might have been considered for NAFLD patients who were likely to have NASH. The findings may thus not represent NAFLD patients in the wider community. However, this would introduce a negative bias, as NAFLD patients in the community would be likely to have milder liver disease, thus increasing the NPV of the FIB4 index. We also acknowledge that pathologic diagnosis was mainly determined using liver tissues derived from percutaneous liver biopsies, which are prone to sampling errors or interobserver variability [7,8]. As recent studies suggest that low normal ALT value does not guarantee freedom from underlying NASH with advanced fibrosis [35-37], it remains to be solved whether FIB4 index can be useful for predicting advanced fibrosis in NAFLD subjects with normal ALT. According to our preliminary data by JSG-NAFLD, the AUROC of FIB4 was 0.810 for the diagnosis of advanced fibrosis in 187 biopsy-proven NAFLD patients with normal ALT levels (data not shown). Our data support the hypothesis that FIB4 index could also be used in the Japanese NAFLD population with normal ALT.

## Conclusion

The FIB4 index demonstrated a good NPV for excluding advanced fibrosis in Japanese NAFLD patients, and could thus be used to reduce the burden of liver biopsies. Larger Asian studies are required to validate the high cut-off point of the FIB4 index. However, the FIB4 test also has several serious limitations, in common with other noninvasive tests for fibrosis, and further research is needed before simple noninvasive tests, including the FIB4 test, can replace liver biopsies in the vast majority of patients.

## Abbreviations

AAR: AST/ALT ratio; AST: aspartate aminotransferase; ALT: alanine aminotransferase; AP index: age to platelet index; APRI- aspartate aminotransferase to platelet ratio index; AUROC: area under the receiver operating characteristic; NAFLD: nonalcoholic fatty liver disease; NASH: nonalcoholic steatohepatitis; NFS: NAFLD fibrosis score; NPV: negative predictive value; PPV: positive predictive value.

## Competing interests

The authors declare that there is no duality of interest associated with this manuscript.

## Authors' contributions

YS: study concept and design, drafting of the manuscript, MY: acquisition of data, HH: acquisition of data, YI: critical revision of the manuscript for important intellectual content. MO: study concept and design, HF: acquisition of data, YE: acquisition of data, YS: acquisition of data, NA: statistical analysis, KK: critical revision of the manuscript for important intellectual content, KF: acquisition of data, KC: critical revision of the manuscript for important intellectual content TS: critical revision of the manuscript for important intellectual content NK: critical revision of the manuscript for important intellectual content KF: critical revision of the manuscript for important intellectual content YK: critical revision of the manuscript for important intellectual content, TY: critical revision of the manuscript for important intellectual content, TO: study supervision, JSG-NAFLD: acquisition of data, study supervision. All authors read and approved the final manuscript.

## Pre-publication history

The pre-publication history for this paper can be accessed here:

http://www.biomedcentral.com/1471-230X/12/2/prepub
